# Analogical modeling: a strategy for developing theories in psychology

**DOI:** 10.3389/fpsyg.2013.00348

**Published:** 2013-06-17

**Authors:** Brian D. Haig

**Affiliations:** Department of Psychology, University of CanterburyChristchurch, New Zealand

The value of analogical modeling in scientific theory construction has been endorsed by a number of philosophers of science (e.g., Hesse, 1966; Harré, 1976; Abrantes, 1999). This article provides a brief overview of the important, but neglected, research strategy of analogical modeling. The strategy has proved to be a valuable resource for the development of many scientific theories. It can be a valuable resource for developing psychological theories as well.

## Analogical modeling

The need for analogical modeling stems from two features that are often present in the initial generation of scientific theories. First, as with exploratory factor analysis, for example, the generation of theories takes the form of explanatory reasoning known as *existential abduction*, through which the existence, but not the nature, of theoretical entities is postulated. In these situations, a suitable research strategy is required in order to learn about the nature of these hidden entities. Analogical modeling is an appropriate strategy for doing the required elaborative work. Second, the postulation of theoretical entities through existential abduction confers an assessment of initial plausibility on those postulations. However, for claims about those latent entities to have the status of genuine knowledge, further evaluative work has to be done. The construction of appropriate analogical models serves to assess further, the plausibility of the expanded understanding they afford, as well as to expand our understanding of those entities.

In science, increasing our knowledge of the nature of our theories' causal mechanisms by analogical modeling is achieved by using the pragmatic strategy of conceiving of these unknown mechanisms in terms of what is already familiar and well understood. Well-known examples of models that have resulted from using this strategy are the model of chromosomal inheritance, based on an analogy with a string of beads; the model of natural selection, based on an analogy with artificial selection; and computational models of the mind, based on analogies with the computer. With respect to the objects of scientific investigation, the strategy of analogical modeling can be represented in the following sequence:

PHENOMENA *are produced by* CAUSAL ENTITIES, *which are represented by* ANALOGICAL MODELS OF CAUSAL MECHANISMS *which lead to* THEORIES CONTAINING DEVELOPED ANALOGICAL MODELS.

In order to understand the nature of analogical modeling further, it is necessary to distinguish between a model, the source of the model, and the subject, or target, of the model (Hesse, 1966; Harré, 1976). From the known nature and behavior of the source, one builds an analogical model of the unknown subject or causal mechanism. To take a biological example, Darwin fashioned his model of the subject of natural selection by reasoning by analogy from the source of the known nature and behavior of the process of artificial selection. In this way, analogical models play an important creative role in theory development.

However, this creative role requires the source from which the model is drawn to be different from the subject that is modeled. For example, the modern computer is a well-known source for the modeling of human cognition, although our cognitive apparatus is not generally thought to be a real computer. Models in which the source and the subject are different are sometimes called *paramorphs*. This is a requirement for the analogical modeling of real and imagined processes. Models in which the source and the subject are the same are sometimes called *homeomorphs*. For example, a toy aeroplane can be a homeomorphic model of a real aircraft. The paramorph can be an iconic representation of real or imagined things. Iconic representation combines elements of visualizable and propositional information in a picture-statement complex that ultimately can be expressed in sentences. The idea of the field of potential in physics is a good example because it is represented in both graphical and sentential form.

It is *iconic paramorphs* that feature centrally in the creative process of theory development through analogical modeling. Iconic models are constructed as representations of reality, real, or imagined. They stand in for the hypothesized causal mechanisms. Although they are representations, iconic models are themselves things, structures, or processes that correspond in some way to things, structures, or processes that are the objects of modeling. They are, therefore, the sorts of things sentences can be about (Harré, 1976). Here we should note that scientific theories that are models represent the world less directly than theories that are not models.

In addition to developing nascent theories, the strategy of analogical modeling also serves to assess their plausibility. In evaluating the aptness of an analogical model, the analogy between its source and subject must be assessed, and for this one needs to consider the structure of analogies. The structure of analogies in models comprises a positive analogy in which the source and subject are alike, a negative analogy in which the source and subject are unlike, and a neutral analogy where we have no reliable knowledge about matched attributes in the source and subject of the model (Hesse, 1966). The negative analogy is irrelevant for purposes of analogical modeling. Because we are essentially ignorant of the nature of the hypothetical mechanism of the subject apart from our knowledge of the source of the model, we are unable to specify any negative analogy between the model and the mechanism being modeled. Thus, in considering the plausibility of an analogical model, one considers the balance of the positive and neutral analogies (Harré, 1976). This is where the relevance of the source for the model is spelled out. Generally speaking, the analogical reasoning scientists employ is informal, and is based upon plausibility arguments.

## Analogical abduction

Reasoning by analogy is an important form of inference, but it is difficult to characterize precisely. Because analogical reasoning results in new knowledge claims, it is ampliative, or content-increasing, a feature it shares with inductive reasoning. However, unlike arguments based on inductive inference, arguments based on analogy can produce knowledge claims about new kinds of things. Briefly, we may say that an analogy is an argument based on assumed, or known, parallels or similarities between two or more objects, properties, or events. What is known about one class of entities is employed to learn more about the other class of entities. A good analogical argument provides an understanding of the less familiar in terms of the more familiar by discerning that the two are alike in relevant respects, but not in other respects. As already mentioned, reasoning by analogy from the known functioning of computers to the less well known character of human cognitive processes is frequently undertaken in psychological research.

Analogical reasoning is important in science and obviously lies at the inferential heart of analogical modeling. It should be emphasized that abductive, or explanatory, reasoning, is an important form of scientific reasoning in its own right. Because the theories fashioned in science are often explanatory theories, the use of analogical modeling in order to develop those theories will necessarily involve combining the two forms of reasoning to produce a creative form of reasoning known as *analogical abduction*. Science often seeks to improve the quality of an explanatory theory by appealing to a similar type of explanation that is known and accepted by the scientific community. In this way, analogical reasoning of an abductive kind is employed.

The reasoning involved in analogical abduction can be simply stated in the form of a general argument schema as follows:

Hypothesis H about property Q was correct in situation S1.
Situation S1 is like the situation S2 in relevant respects.
Therefore, an analog of H might be appropriate in situation S2.

Darwin's (1958) theory or model of natural selection, and the other aforementioned analogical models, make essential use of analogical abduction. The general argument for analogical abduction just given can be rewritten in simplified form for Darwin's case follows:

The hypothesis of evolution by artificial selection was correct in cases of selective domestic breeding.
Cases of selective domestic breeding are like cases of the natural evolution of species with respect to the selection process.
Therefore, by analogy with the hypothesis of artificial selection, the hypothesis of natural selection might be appropriate in situations where variants are not deliberately selected for.

In formulating his theory of natural selection, Darwin took advantage of the two most important features of analogical abduction: Its ability to create, and its ability to justify. In reasoning by analogy, using known facts about artificial selection, Darwin was able to hypothesize the parallel mechanism of natural selection that explained diversity among natural species. At the same time, he was able to appeal to the epistemic worth of his carefully crafted analogy and proclaim the plausibility of his hypothesis of natural selection. Numerous creative scientists have been able to exploit the resources of analogical abduction in this manner.

## The dramaturgical model

An instructive example of an analogical model in psychology is Rom Harré's (1979) role-rule model of microsocial interaction. With the role-rule model, Irving Goffman's (1959) dramaturgical perspective on human action provides the source model for understanding the underlying causal mechanisms involved in the production of ceremonial, argumentative, and other forms of social interaction. This model too can be presented in accordance with the simple argument schema used immediately above in order to display the bare-bones structure of its analogical abductive reasoning:

The theory of dramaturgy provides a correct account of behavior on the theatrical stage.
Behavior on the theatrical stage is like a good deal of human behavior in social life.
Therefore, by analogy with the theory of dramaturgy, much human social behavior might be understood and monitored as actors on life's stage.

The basic idea of the dramaturgical perspective is that we observe and hear a simulacrum of life on the stage, and that our knowledge of how this is produced provides us with a guide to the creation of real life. Goffman's dramaturgical perspective provides a detailed analytical account of the roles and rules human agents follow on life's stage combined with a “watchful consciousness” of the actor, the producer, the audience, and the critic.

As a source model, the dramaturgical model has both positive and negative analogies for there are clear similarities and differences between real life and dramatically staged acts. Regarding similarities, Goffman noted that to be understood as the person they are portraying, the actor has to act in manner that parallels what the audience would expect of that kind of person. Clearly, there are differences between stage drama and real life. The differences involve sequences of acts and actions that are at once selective, simplified, and heightened. For example, in comparison with real life, only a limited number life sequences are followed, time is compressed, and resolutions are effectively reached (Harré, 1979). The reduction in the number of life sequences and the compression of time are abstractive processes. The use of successful resolutions is an idealized move. In these ways, the modeling strategies of abstraction and idealization are employed to simplify the complex domain of microsocial interaction.

## Conclusion

Analogical modeling in science is a risky cognitive undertaking. In practice, it involves reading widely in neighboring disciplines for potentially fruitful source models, and selecting those source models that have worked well in theories that are judged similar to the theory being developed. In addition, the methodology of modeling through analogical abduction provides useful guidance for expanding scientists' knowledge of latent causal mechanisms (see, e.g., Harré, 2004; Haig, 2014). Of course, the relevant limits of the similarity relation between the source and subject of the model are decided with reference to contingent matters of fact that are specific to particular cases, not by reference to general advice on analogical modeling. Unlike psychology's modal research practice of evaluating hypotheses via simple hypothetico-deductive testing, the evaluation of analogical models involves making plausibility judgments about them. However, these judgments need to be strengthened by subsequently comparing the developed theories to their rivals before the models can be regarded as properly credentialed. Researchers who want to engage in analogical modeling will have to adopt more of a do-it-yourself attitude than is the custom in psychology.
